# Local anesthetic thoracoscopy for the diagnosis of metastatic pleural melanoma originated from oral malignant melanoma: case report and comments

**DOI:** 10.1186/s12957-015-0741-0

**Published:** 2015-12-01

**Authors:** Bingjun Yang, Qingzhao Li, Hui Zhao, Huibin Liu, Tao Tang, Chunyang Jiang

**Affiliations:** Department of Thoracic Surgery, Tianjin Union Medicine Centre, 190 Jieyuan Road, Hongqiao District, Tianjin 300121 People’s Republic of China; School of Public Health, North China University of Science and Technology, Tangshan, 063001 Hebei People’s Republic of China; Office of Clinical Drug Trial Institution, The Affiliated Tumor Hospital of Xinjiang Medical University, Urumqi, Xinjiang 830011 People’s Republic of China; Department of Pathology, Tianjin Union Medicine Centre, 190 Jieyuan Road, Hongqiao District, Tianjin 300121 People’s Republic of China

**Keywords:** Pleural melanoma, Thoracoscopy, Oral malignant melanoma, Local anesthesia, Diagnosis

## Abstract

**Background:**

Oral malignant melanoma (OMM) is an aggressive tumor with very low survival rate and easy to metastasize. Pleural metastatic melanoma via primary OMM is rare.

**Case presentation:**

In this report, we presented a case of metastatic malignant melanoma of the pleura originated from OMM. A 54-year-old man without primary skin lesion was diagnosed multiple nodular shadows, pleural invasion, and pleural effusion by chest computed tomography (CT). One cyst-form tumor on the tongue base was observed by bronchoscopy, which was diagnosed as OMM by pathological examination and then was resected. After getting the tumor tissues from the pleura by pleural biopsy surgery, the diagnosis of pathological examination was pleural metastatic melanoma. Furthermore, tumor cells displayed a positive immunoreaction for melanocytic markers S100 and HMB-45 combining with positive vimentin and cytokeratin AE1/AE3. The patient was therefore diagnosed with metastatic melanoma of the left pleura and the primary melanoma was OMM.

**Conclusions:**

According to this case, we could draw the conclusion that pleural metastasis from OMM was very rare and thoracoscopy preceded under local anesthesia is an important method for its accurate diagnosis.

**Electronic supplementary material:**

The online version of this article (doi:10.1186/s12957-015-0741-0) contains supplementary material, which is available to authorized users.

## Background

Melanomas, including two subtypes, are originated from melanoblasts or melanocytes which can have a benign or malignant clinical course. They mainly occur on the skin and are relevant to ultraviolet light exposure as common tumors in human [1]. The incidence of melanoma is increasing about 5 % per year worldwide [2]. Malignant melanoma (MM) is the most aggressive skin cancer originating from melanocytes with a high degree of phenotypic plasticity, which is primarily located in the skin but also found in eyes, ears, mouth, gastrointestinal tract, genital mucosa, and leptomeninges [3]. Although melanoma accounts for only 5 % of all malignant skin tumors, it has occupied 75 % deaths of all skin tumor patients [4]. Surgery can be performed as an effective treatment at the early phase of the tumor. Unfortunately, melanoma is prone to metastasis by lymphatic pathway to regional lymph nodes and even by circulation to distant sites, which can significantly worsen the prognosis [5, 6]. The median survival time for metastatic melanoma patients is only about 6–9 months [7, 8].

Primary melanoma of the thorax, which mainly involves the lung and pleura, is exceptionally rare. In this report, we presented a case that was diagnosed as metastatic MM of the pleura by pleural biopsy surgery, which was originated from an oral malignant melanoma (OMM). The initial symptoms of this patient were chest pain and dyspnea for 2 months. Chest computed tomography (CT) which was performed on a 64-row multidetector CT scanner (Somatom Sensation, Siemens, Erlangen, Germany) showed multiple nodular shadows, pleural invasion, and pleural effusion, and at first, the case was misdiagnosed as lung and pleural metastatic carcinoma. Therefore, thoracoscopy, including video-assisted thoracoscopic surgery (VATS) and simple rigid thoracoscopy, is the key to the accurate diagnosis of this disease.

## Case presentation

**Fig. 1:**
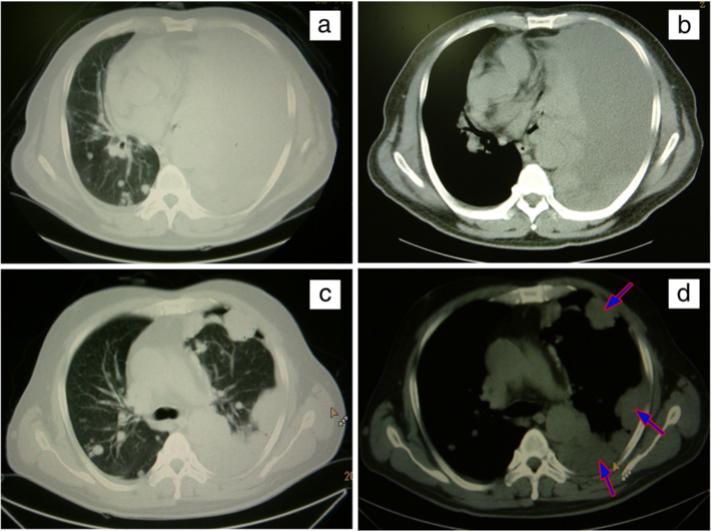
The chest CT imaging of the pleural effusion and lesions. Before operation, pleural effusion and lesions in the left thorax and multiple metastases in the right lung can be seen in CT scan imaging (**a** and **b**). After complete drainage of pleural effusion, metastatic neoplasms in pleura, enlargement of mediastinal lymph nodes, and lung metastases were clearly emerged by countercheck CT scan (**c** and **d**). The pleural lesions were pointed by *blue arrows* in **d**. **a** and **c** Lung window. **b** and **d** Mediastinum window

**Fig. 2:**
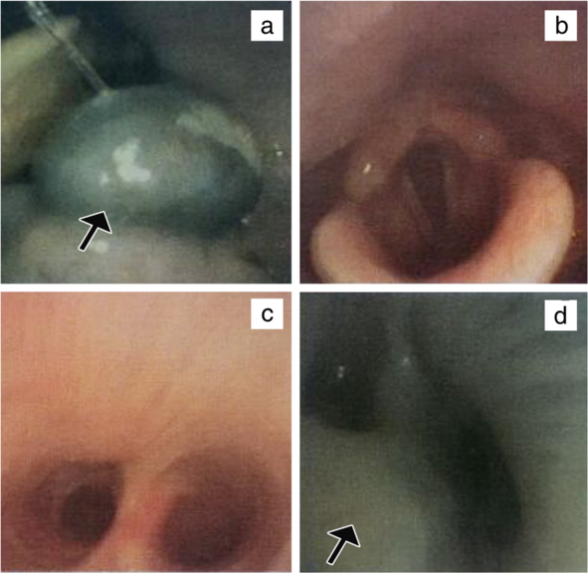
NBI bronchoscopy. One cystoma was on the left side of the tongue base (**a**). The cystoma was observed by bronchoscopy and pointed by *black arrows*. Abnormalities were not seen in the glottis and tracheal juga (**b** and **c**). Bronchial stenosis of left pulmonary segments can be seen in **d**

**Fig. 3:**
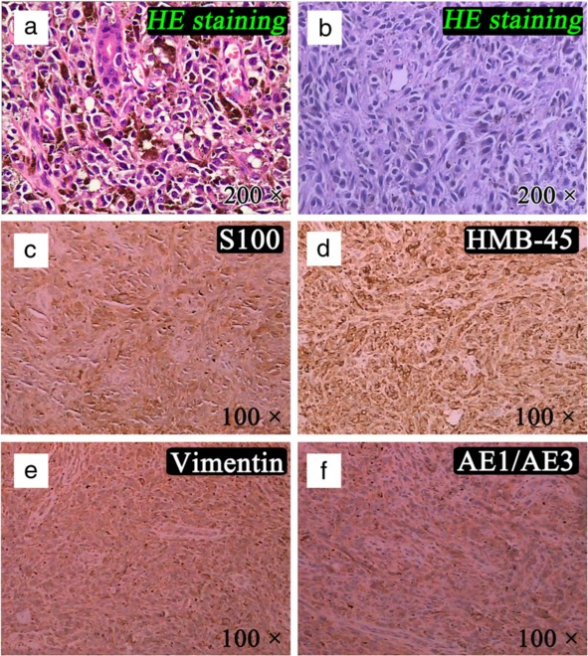
The pathological results of the resected tumor from the tongue base and pleural tumor tissue and immunohistochemical detection of the pleural tumor tissue. The tissue’s histopathology changes were observed in light microscope (Nikon Eclipse 80i, Tokyo, Japan) and photos were taken. Postoperative pathological results for the tongue base and pleural tumor tissue, respectively (**a** and **b**) (×200). The immunohistochemical results of the pleural tumor tissue are shown in **c**–**f**, which are S100, HMB-45, vimentin, and cytokeratin AE1/AE3, respectively, and the magnification are all of ×100

The patient was a 54-year-old Han Chinese man who, in June 2013, was diagnosed as double-sided pleural effusion which was combined with the left multifocal pleural lesions and multiple lung metastases in the right lung by CT scan (Fig. 1a, b). The previous medical history reported no primary skin lesion including melanin stain. The related examines were finished before thoracoscopy pleural biopsy operation. One cyst of the neoplasm on the left side of the tongue base and multiple bronchial stenosis of left pulmonary segments were observed by narrow band imaging (NBI) bronchoscopy (Olympus, EVIS LUCERA) (Fig. 2). The tumor was then resected and diagnosed as OMM by pathological examination (Fig. 3a) and the immunohistochemical staining results (Additional file 1: Figure S1). The results of tuberculosis antibody (TB-Ab) and TB-DNA in serum were all negative. Serum tumor markers for lung carcinoma including carcino-embryonic antigen, carbohydrate antigen 72-4, squamous cell carcinoma, cyfra 21-1, cytokeratin 19 fragments, and ferritin were all in normal range. However, carbohydrate antigen 125 and neuron-specific enolase were about three times and two times higher than the upper limits, respectively.

**Fig. 4:**
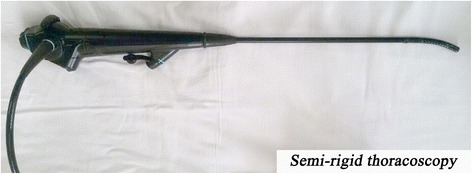
The semi-rigid thoracoscopy was used in pleural biopsy surgery

**Fig. 5:**
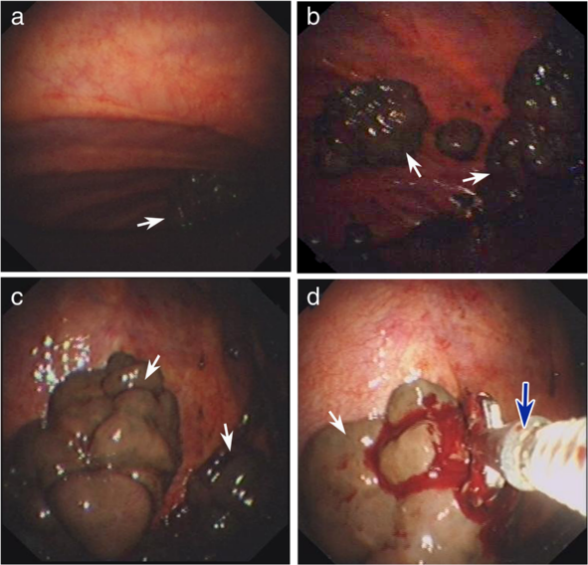
The main observation of pleural biopsy surgery in the right parietal pleura. Multiple violet-black neoplasms can be seen in the parietal pleura by thoracoscopy with video assistance, being different sizes, very brittle, and prone to bleeding, and these lesions are pointed by *white arrows* (**a**–**c**). **d** The tumor tissues were gained by biopsy forceps. The used forceps is pointed by *blue arrow*

Subsequently, the pleural biopsy surgery of the left thorax was preceded by semi-rigid thoracoscopy (model LTF-240, Olympus, Tokyo, Japan) (Fig. 4) with video assistance under local anesthesia using 10 mL 1 % Lidocaine injected into the subcutaneous tissue. The thoracoscopy was entered from a single port in the sixth intercostal space. After extraction of 1500 mL bloody pleural fluid, multiple violet black neoplasms can be seen in the parietal pleura, which were different sizes, very brittle, and prone to bleeding (Fig. 5). After biting from several sections of these lesions, the tissues were used for pathological detection. Pleural effusion was continuously discharged by using closed drainage tube. Cytology examination of pleural effusion displayed red blood cells in the field of vision and severe abnormity of cell nucleus.

Postoperative pathological reports showed that these neoplasms were pleural metastatic melanoma by hematoxylin-eosin (HE) staining, and combined immunohistochemical results suggested that the HMB-45, S-100, vimentin, and AE1/AE3 (antibodies were all purchased from Santa Cruz Biotechnology (Santa Cruz, CA)) were all significantly positive expressions (Fig. 3b–f).

After almost complete drainage of pleural effusion, remarkable multiple metastatic neoplasms in the pleura, enlargement of mediastinal lymph nodes, and several lung metastases could be clearly observed by countercheck CT scan (Fig. 1c, d). During the inpatient period, detections of brain CT scan, abdominal Doppler ultrasound, and so on, there were no evidence to confirm other organs and any other lymphatic node metastases. Subsequently, there was no further treatment, and less than 6 months of follow-up, the patient died.

This case report and related experimental protocols were approved by the ethics committee of Tianjin Union Medicine Centre of China.

### Discussion

In the past several decades, the incidence of melanoma has been steadily rising with an annual increase of 3–8 % worldwide [9]. The most common form of melanoma is the cutaneous or the ocular form. As neural crest-derived cells, melanocytes could migrate to the skin, mucous membranes, and other sites. Melanoma metastasizing to thorax is common, but primary pulmonary or pleural melanoma is extremely rare. Therefore, MM has very seldom been described as a primary tumor in the pleura or lower respiratory tract, and there are only very limited literatures that have been reported [10]. Since metastasis of MM to the lung or pleura is relatively common, it is very important to distinguish primary from secondary melanomas.

OMM, which was first described by Weber in 1859, is a much rare neoplasm located at the basal layer of the oral mucous membranes owing to the uncontrolled growth of melanocytes [11]. Mucosal melanoma accounts for only 0.5 % of all oral tumor involving the sinonasal cavity, oral cavity, pharynx, larynx, and upper esophagus [12]. Oral melanomas occur slightly more often in males (2.8:1, male to female ratio) and with an average age of 56 years (the age range is from 20 to 83 years) [13]. OMM belongs to head and neck mucosal melanomas (HNMM) and frequently exhibits postoperative recurrence and distant metastasis. For HNMM patients, surgery is recommended if indicated, and surgery combined with postoperative radiotherapy is also recommended for dramatically improved local control of the tumor bed. Radiotherapy and immunological therapy could be potential options for patients without surgery chance [14].

In the present case, the primary site of melanoma was previously found in the root of the tongue without primary skin lesion. After obtaining the tumor tissues from several sites of parietal pleura by pleural biopsy surgery, the diagnosis of pathological examination was pleural metastatic melanoma through HE staining method. Furthermore, tumor cells displayed a positive immunoreaction for melanocytic markers (S100 and HMB-45, which are frequently expressed in primary oral melanomas and helpful to confirm the diagnosis [15]). In addition, both vimentin and cytokeratin AE1/AE3 also appeared as positive expressions. We therefore ascertained that the metastatic melanoma of the left pleura was metastasized from OMM.

Modern thoracoscopy provides a potentially less invasive means to diagnose and to treat a variety of intrathoracic diseases. Simple rigid thoracoscopy or VATS is safe and effective for the diagnosis of both benign and malignant pleural disease which has very high sensitivity (80 to 100 %) [16]. For the present patient, the color and the appearance of the melanoma are easy to recognize under thoracoscopy, with the potential to limit complications and to reduce morbidity and hospital stays for thoracotomy. Pleural tumorous diseases are one important indication for thoracoscopy with video assistance and can easily be diagnosed accurately.

## Conclusions

We have presented a very rare case of amelanotic melanoma of the left parietal pleura that was diagnosed as a metastatic tumor from OMM by detailed physical examination and pathological and immunohistochemistry detection. Pleural metastasis via OMM was very rare, and thoracoscopy preceded under local anesthesia is an effective and safety method for its accurate diagnosis.

## Consent

The patient and his family members were informed and have consented for the publication of this report. A copy of the written consent is available for review by the Editor-in-Chief of this journal.

## Additional file

Additional file 1: Figure S1. The immunohistochemical results of the primary tongue melanoma tissue are shown in (**a**), (**b**), (**c**) and (**d**), which are S100, HMB-45, vimentin, and cytokeratin AE1/AE3, respectively, and the magnification are all of ×200 (JPG 2042 kb)

## Abbreviations

CT: computed tomography
HE: hematoxylin-eosin
HNMM: head and neck mucosal melanoma
MM: malignant melanoma
NBI: narrow band imaging
OMM: oral malignant melanoma
TB-Ab: tuberculosis antibody
VATS: video-assisted thoracoscopic surgery
