# Supplementary figures and images for: Local anesthetic thoracoscopy for the diagnosis of metastatic pleural melanoma originated from oral malignant melanoma: case report and comments
Source: World J Surg Oncol. 2015 Dec 1;13:326. doi: 10.1186/s12957-015-0741-0 (PMC4666196; doi:10.1186/s12957-015-0741-0)

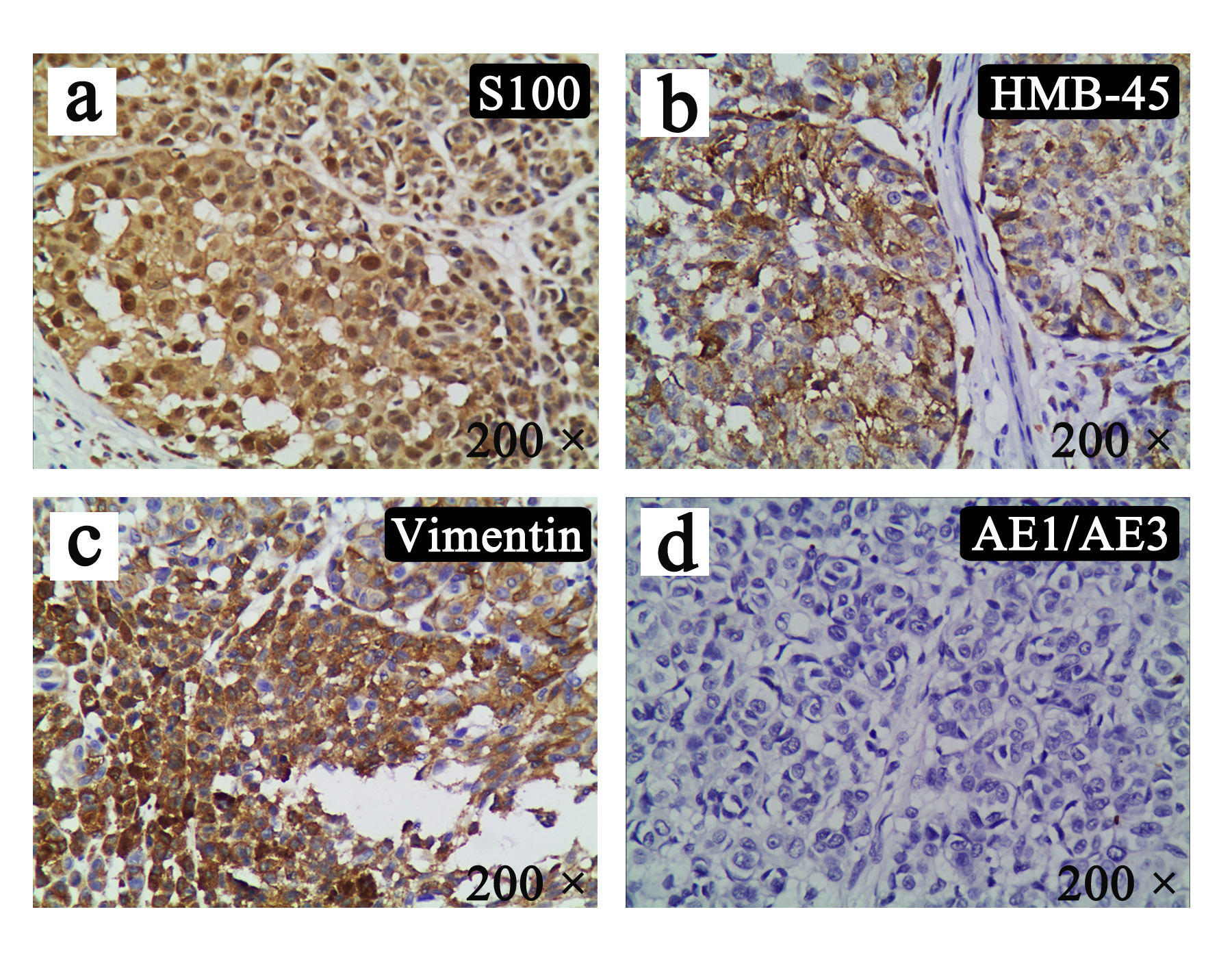

Supplement: Additional file 1: Figure S1. — The immunohistochemical results of the primary tongue melanoma tissue are shown in (a), (b), (c) and (d), which are S100, HMB-45, vimentin, and cytokeratin AE1/AE3, respectively, and the magnification are all of ×200 (JPG 2042 kb) [file 12957_2015_741_MOESM1_ESM.jpg]
